# Orthobiologics and hyaluronic acid usage in the Netherlands: an electronic survey of 265 orthopaedic surgeons and sports physicians

**DOI:** 10.1186/s40634-021-00380-9

**Published:** 2021-08-19

**Authors:** J. J. de Graeff, M. P. J. van den Bekerom, B. L. van Meer, J. A. C. Zijl, On behalf of the Dutch Arthroscopy Society

**Affiliations:** 1 Department of Orthopaedic Surgery, Joint Research, OLVG, Oosterpark 9, 1091 AC Amsterdam, the Netherlands; 2 Department of Orthopaedic Surgery, Leiden University Medical Center, PO Box 9600, 2300 RC Leiden, The Netherlands; 3 Department of Human Movement Sciences, Faculty of Behavioural and Movement Sciences, Amsterdam Movement Sciences, Vrije Universiteit Amsterdam, Van der Boechorststraat 7, 1081 BT Amsterdam, the Netherlands; 4 Department of Sports Medicine, St. Antonius Hospital, Utrecht, The Netherlands; 5 Department of Orthopaedic Surgery, St. Antonius Hospital, Utrecht, The Netherlands

**Keywords:** Orthobiologics, MSCs, Hyaluronic acid, Platelet rich plasma

## Abstract

**Purpose:**

“Biologic therapies” in the field of orthopaedic surgery and sports medicine, so called orthobiologics, have been gaining significant interest from physicians and patients, with increasing usage over the recent years. The aim of this study is to (1) evaluate the usage of orthobiologics in the Netherlands, (2) to clarify the reasons for the use or non-use of orthobiologics, and (3) the most addressed disease for use of orthobiologics.

**Methods:**

The authors created a 19-quenstion online survey comprised of both closed-ended and open-ended response questions in order to examine the use of and the indication for orthobiologics. The survey was sent to all the members of the Dutch Orthopaedic Association and Netherlands Association of Sports Medicine of which 15% responded.

**Results:**

The majority of the 265 respondents (65%) did not treat patients with or refer patients for treatment with orthobiologics. The most important reasons for not using orthobiologics were the lack of scientific evidence, the lack of good experience, and the lack of insurance coverage. Of the physicians that used orthobiologics, the most used hyaluronic acid (76%) and platelet-rich plasma (27%). Orthobiologics were most used for knee osteoarthritis and medial or lateral epicondylitis.

**Conclusion:**

Although some orthobiologic treatments might be effective and the research interest is growing, our study shows that the majority of orthopaedic and sport physician clinicians in the Netherlands still does not make use of orthobiologics as a treatment option, but almost a third of them is contemplating to start using orthobiologics.

**Level of evidence:**

III.

**Supplementary Information:**

The online version contains supplementary material available at 10.1186/s40634-021-00380-9.

## Introduction

“Biologic therapies” in the field of orthopaedic surgery and sports medicine, so called orthobiologics, have been gaining significant interest from physicians and patients, with increasing usage over the recent years. The growing interest is also shown by the recent orthobiologics Initiative (ORBIT) of the ESSKA (European Society for Sports Traumatology, Knee Surgery and Arthroscopy). Orthobiologics are biologically derived materials which aim to promote regeneration of tissues that are the focus of orthopaedic surgery. These biomaterials are naturally present in the human body. Urist et al. was one of the first to describe such a substance over 50 years ago, named bone morphogenic protein (BMP), which would assist bone regeneration [26]. There are many different types of orthobiologics, the most common are autologous blood injection (ABI), platelet-rich plasma (PRP), autologous conditioned serum (ACS), and cell-based therapies (CBT). Within the CBT category there several options such as stromal vascular fraction, Mesenchymal Stromal Cells or Bone Marrow Aspirate Stem Cell Concentrate. In our survey the general term Mesenchymal Stromal Cells (MSC) was used. In some of the literature hyaluronic acid (HA) is also mentioned as an orthobiologic so this therapy is also included in our study. Orthobiologics have been suggested to improve healing and pain subsequent to various cartilage, ligament, tendon, and bone pathologies. These treatments have been popularized based on the inherent safety of autologous products, minimal regulatory obstacles, and strong marketing, despite mixed clinical data [20]. The literature yields mixed results on the benefits of many of these treatments [6, 16, 17, 19, 21, 27].

Although some basic science studies showed promising results of orthobiologics in tissue repair and regeneration and some studies claimed it might inhibit the progression of osteoarthritis, there is no clinical evidence to support this theory [14]. The current rationale of use for ABI, PRP, and ACS is that the injections have positive effects on chondrogenesis and mesenchymal stem cell proliferation [12, 19]. They have also been shown to decrease inflammatory markers and promote anti-inflammatory mediators, as well as reducing the expression of inflammatory enzymes [19, 24].

For MSC the rationale is that it has immunomodulatory properties, which modulates inflammatory responses and prevent immune responses mediated disease [18]. MSC can stimulate angiogenesis, limit inflammation, and recruiting local tissue-specific progenitors [15]. There is also a great and growing commercial interest in orthobiologics over the recent years which has held to active commercial promotion campaign [20]. Hadley et al. studied commercial products promoted at American Orthopaedic Society for Sports Medicine (AOSSM) and the Arthroscopy Association of North America (AANA) Annual Meetings and found that 65% (15 of 23) of the biologic treatments promoted at the AOSSM 2016 and AANA 2017 meetings had no peer reviewed publications directly supporting their use [7].

In the recently published Osteoarthritis Research Society International (OARSI) guidelines the use of intra-articular MSC and PRP were discommended, and intra-articular HA was conditionally recommended in individuals with knee OA, but is not recommended for individuals with hip or polyarticular OA [2]. This was in line with the recommendations of the European Society for Clinical and Economic Aspects of Osteoporosis, Osteoarthritis and Musculoskeletal Diseases (ESCEO) guidelines published the same year [4]. In the Netherlands the Dutch Orthopaedic Association (NOV) recently published a similar statement that HA is not recommended as a primary course of treatment for osteoarthritis of the knee but can be considered a secondary course of treatment. The NOV does not recommend the use of PRP. In the Netherlands most insurances do not cover the use of orthobiologics.

In the light of a worldwide growing interest and usage of orthobiologics the aim of this study is to (1) evaluate the usage of orthobiologics in the Netherlands, (2) to clarify the reasons for the use or non-use of orthobiologics, and (3) the indications of use for orthobiologics.

## Materials and methods

### Participants and setting

Between February 2020 and July 2020, an online survey was conducted among orthopaedic surgeons, orthopaedic surgery residents, sport physicians, and sport physician residents in the Netherlands. The design and report of our study followed the guidelines for reporting medical surveys outlined by Artino et al. [1] In May 2020 orthopaedic surgeons and orthopaedic surgery residents were invited by the NOV on behalf of the Dutch Arthroscopy Society (NVA) to fill out the digital survey. The first invitation to the sport physicians and sport physician residents was sent by the Netherlands Association of Sports Medicine (VSG) in February 2020 and a reminder was sent in June 2020. The survey was constructed and distributed using an online survey platform (Survey Monkey). Responses were collected confidentially. Ethics approval was not required.

Sample size was calculated using the Raosoft® software at the developer’s website. The required sample size was estimated at 90% confidence level with an estimated 50% response distribution and a margin of error of ±5%. The required minimum sample size was determined to be 236 [5]. The population size was based on the total of members of the NOV and NVA at the time of the survey (*n* = 1828).

### Survey

The authors created a 19-quenstion Dutch survey, comprised of both closed-ended and open-ended response questions in order to examine the use of and the indication for orthobiologics. The respondents were asked to fill out two general questions: the first to identify their profession (orthopaedic surgeon, orthopaedic surgery resident, sport physician, sport physician resident or other, which had to be specified) and the second to identify the frequency of cases where they treated a patient with or referred a patient for treatment with orthobiologics (daily weekly, monthly, yearly or never). If the respondents never treated with or referred a patient for treatment with orthobiologics three questions followed: the first to identify the reasons for not using orthobiologics, the second to see if there were contemplating to start using orthobiologics, and the last open-ended question to give the opportunity for remarks or suggestions.

The respondents that use orthobiologics as a treatment answered 14 closed-ended and open-ended response questions to clarify to following study topics: the type of orthobiologics they used (ABI, PRP, ABI, MSC, HA, or “other” which had to be specified); the frequency and dosage of use; the indications for use; and the physician’s satisfaction regarding the treatment.

### Statistical analysis

Frequencies were calculated for each closed-ended question on the survey. The chi-square test was used to explore potential associations between selected categorical variables. Among respondents two sub-analyses were conducted to compare Orthopaedic surgery physicians (surgeons and residents) versus sport physicians, and non-residents versus residents. We used STATA 13.0 (StataCorp LP, college station, TX) to perform our statistical analyses, and considered *P* values of < 0.05 to be significant.

## Results

Our survey was sent to all 1457 members of the NOV and 371 members of the VSG. Of all recipients 275 responded to our questionnaire (15%). Of the 275 respondents 198 (72%) were orthopaedic surgeons, 35 (12,7%) were orthopaedic surgery residents, 24 (8,7%) were sport physicians, 2 (0,7%) were sport physicians in training, and 6 had other medical professions (2,2%). Ten respondents (3,7%) were dropped from the further analysis because they were no longer practicing orthopaedic surgery (*n* = 8) or had no clinical activities (researchers, *n* = 2).

Of the remaining 265 respondents, 35,1% (*n* = 93) used or referred patients for treatment with orthobiologics or HA. There was no statistical difference in use or non-use between Orthopaedic surgery physicians versus sport physicians (*X*^2^ = 2.96, *p* = 0.085) or non-residents versus residents (*X*^2^ = 0.54, *p* = 0.461).

Table 1 shows the frequency of use or referral, the type of treatment that are being used, the reasons for use, and the satisfaction of the effect according to the physician. The indications for use are show in Table 2, broken down into the different locations of use.

**Table 1:** The use of orthobiologics

	n (%)
**Use (*n*** **= 265 respondents)**	
*Never*	172 (64,9)
*Yearly*	44 (16,6)
*Monthly*	25 (9,4)
*Weekly*	18 (6,8)
*Daily*	6 (2,3)
**Type (*n*** **= 67 respondents)** ^**ab**^	
*Hyaluronic acid*	51 (76,1)
*Platelet-rich plasma*	18 (26,9)
*Autologous blood injection*	11 (16,4)
*Autologous conditioned serum*	7 (10,4)
*Bone matrix*	5 (7,5)
*Mesenchymal stem cells*	4 (6,0)
**Reason of use**^**b**^	
*Effective treatment*	37 (55,2)
*Postponing surgical treatment*	30 (44,8)
*Placebo effect*	12 (17,9)
*Last resort*	5 (7,5)
*Research purposes*	3 (4,5)
**Satisfaction physician**^**b**^	
*Very satisfied*	2 (3,0)
*Satisfied*	32 (47,7)
*Neutral*	29 (43,3)
*Unsatisfied*	1 (1,5)
*Very unsatisfied*	2 (3,0)
*Unanswered*	1 (1,5)

^a^ of the 93 respondents that were using orthobiologics, 67 (72%) filled in the follow up questions specifying their use

^b^ Multiple answers possible

**Table 2:** Indications for use of orthobiologics ^a^ (*n* = 67)

	n (%)
**Shoulder**	
*Omarthrosis*	9 (13,4)
*Peroperative during cuff repair*	4 (6,0)
*Rotator cuff tendinopathy*	4 (6,0)
*Subacromial bursitis*	1 (1,5)
*Pseudoarthrosis / non-union repair*	1 (1,5)
**Elbow**	
*Lateral epicondylitis*	19 (28,4)
*Medial epicondylitis*	12 (17,9)
*Biceps tendinopathy*	7 (10,5)
*Collateral ulnar ligamentum*	6 (9,0)
*Elbow arthrosis*	1 (1,5)
**Hip**	
*Osteoarthritis of the hip*	4 (6,0)
*Avascular necrosis femur head*	1 (1,5)
*Gluteal tendinopathy*	1 (1,5)
*Hip impingement*	1 (1,5)
**Knee**	
*Osteoarthritis of the knee*	63 (94,0)
*Jumper’s knee*	15 (22,4)
*During ACL reconstruction*	5 (7,5)
*During cartilage transplant*	1 (1,5)
*Osteochondral defect*	1 (1,5)
*Bursitis*	1 (1,5)
**Foot/Ankle**	
*Achilles tendinopathy*	13 (19,4)
*Plantar fasciitis*	12 (17,9)
*Ankle arthrosis*	3 (4,5)
*Osteochondral defect*	3 (4,5)
*During Achilles tendon repair*	1 (1,5)
**Other**	
*Non-union treatment*	3 (4,5)
*Chronic osteomyelitis treatment*	1 (1,5)

^a^ Multiple answers possible

Of the different treatments HA is used the most (76,1%). Orthopaedic physicians more often used or referred patients for treatment with HA than sport physicians (*X*^2^ = 8.35, *p* = 0.004), whereas sport physicians more often used or referred patients for treatment with ACS (*X*^2^ = 6.85, *p* = 0.009). For the other treatments there were no statistical differences. Between non-residents versus residents, there was no statistical difference in use of the different discussed treatments.

The most important reason of physicians for not using orthobiologics or HA (*n* = 172) were the lack of scientific evidence (45,3%), the lack of good experience (39,5%), and the lack of insurance coverage (1,8%).

Almost a third of the physicians (32,6%) not using or referring patient for treatment with orthobiologics or HA, is contemplating to start using orthobiologics.

## Discussion

The main finding of this study was the majority of the 265 respondents (64,9%) among Dutch orthopaedic surgeons and sports physicians does not treat patients with or refer patients for treatment with orthobiologics or HA. The most important reason of physicians for never using orthobiologics are the lack of scientific evidence (45,3%), and the lack of good experience (39,5%). The relatively low percentage of use might fit with the inconclusive literature. The most used treatments were HA and PRP (respectively 76,1% and 26,9%) and the most frequent mentioned indications for the use were osteoarthritis of the knee (94,0%), a jumpers knee (22,4%), and lateral and medial epicondylitis (respectively 28,4 and 17,9%).

In line with our findings the literature shows that HA is one of the most used orthobiologics used for OA of the knee [29]. The rationale of HA relies on two mechanisms: first, as a supplement to intra-articular mechanical viscosity, it can play the role of joint protection such as lubrication, shock absorption and friction reduction; Second, the homeostasis of joint is reconstructed by guiding the secretion of endogenous HA [29]. In comparison to intra-articular injection of corticosteroids Rodriguez-Merchan showed that intra-articular injections of HA provided a more lasting relief. He found corticosteroids showed a significant clinical effect only in the first 4 weeks. However, the effect provided by HA lasted for 26 weeks [23]. It also suggested that HA has fewer side effects than corticosteroids for patients with osteoarthritis [3, 8]. For OA in other joint the literature shows no significant benefit for treatment with HA. A systematic review of Gazendam et al. concluded that the current evidence suggests that intraarticular hip saline injections performed as well as PRP or HA in the management of hip pain and functional outcomes [6]. The use of HA in tendinopathy remains highly debated. Some in vitro and animal in vivo studies demonstrate encouraging results, but clinical ramifications remain unsure [13]. A meta-analysis studying the use of HA in rotator cuff tendinopathy showed no significant functional improvement or pain reduction at short (3–6 weeks), medium (12 weeks) or long term (24 weeks) [17]. In line with OARSI and ESCEO guidelines the NOV considers HA a secondary course of treatment for knee OA, but is not recommended for individuals with hip or polyarticular OA [2, 4]. A recent study of Hermans et al. showed that intra-articular HA injections had a good effect for knee OA in Dutch patients of working age and suggested that the use of HA is cost-effective for the Dutch health care situation [9, 10].

PRP is produced by centrifugation (“spinning down”) of a small sample of the patient’s blood to isolate and concentrate the platelets. Both platelets and plasma (the fluid portion of blood) contain a number of proteins that can potentially decrease inflammation, improve pain, and aid in tissue healing. PRP has been used for many soft tissue injuries, including ligament, tendon, meniscus, cartilage, and muscle injuries. The most common indications for use are: knee OA, Jumpers knee and epicondylitis of the elbow [22]. A study by Xing et al. which reviewed 10 systematic reviews concerning PRP injections for knee OA concluded PRP is an effective intervention in treating knee OA without increased risk of adverse events [28]. Lateral and medial epicondylitis are also frequently treated with PRP and ABI injections. A recent meta-analysis comparing corticosteroid (CSI), ABI, and PRP injections showed that CSI improves functional outcomes and pain relief in the short term (< 12 weeks), while AB and PRP are the most effective treatments in the intermediate term (12–26 weeks) [11]. A recently published statement of a German working group consisting of orthopaedic- and trauma surgeons stated that PRP as therapy for cartilage, tendon and muscle damage was regarded useful and its importance might grow it the future. The application of PRP for early knee osteoarthritis (Kellgren-Lawrence grade I/II) was regarded as potentially useful, as well as for acute and chronic tendinopathies. For chronic lesions (cartilage, tendons), multiple injections (2–4) were seen preferable to singular injections. They called for further standardization of the PRP preparation methods, indication and application protocols for knee osteoarthritis and other indications, which should be further evaluated in basic science studies and randomized controlled clinical trials [25]. The Orthopaedic Research Society (ORS), American Academy of Orthopaedic Surgeons (AAOS) and the NOV do not advise as a standard treatment with PRP for the above mentioned illnesses.

The clinical studies for treatment with MSCs are scarce and focusses mainly on the treatment of osteochondral defects, OA and tendinopathy. Treatment with MSC holds a great potential because they have the potential to contribute to tissue regeneration directly by differentiation into damaged cell types or indirectly by stimulating angiogenesis, limiting inflammation, and recruiting local tissue-specific progenitors, but there is no high-quality evidence available at this time to support the routine use of stem cell therapy for any musculoskeletal injuries [15]. The apparent lack of use of MSC in the Netherlands is notable. This might be because it is not part of the standard treatment guidelines and is often only used in a research setting.

Our study has several limitations. Firstly, a general limitation attributed to this survey research is the possible oversimplification of the studied topic by studying the use of orthobiologics in general. Ideally the survey would have been contracted using Likert scale response options but due to variety of orthobiologic types questioned and for the sake of simplicity and pragmatism multiple choice questions were used. Also, it is hard to meticulously analyse the use of specific types of orthobiologics due to the lack of clear practice guidelines and the difference of concentrations of product. Moreover, due to the potential oversimplification it might have been unclear to the respondents if the survey was focussed solely on injections with orthobiologics or also on the perioperative use. Secondly, the national aspect of this survey to might be of influence. The differences in availability and coverage of these treatments may vary between countries. Thirdly, an important concern in survey research are the problems related to validity and reliability of results. Even when questions are correctly formulated and well-intentioned, there is always a chance of being unclear or misunderstood. The main strength of our study is the fact it is the first study that evaluates the use and reasons for (non-)use of orthobiologics in daily clinical practice in the Netherlands. Fourthly, almost a third of the respondents who use orthobiologics did not provide specific information about the type of orthobiologic they used.

Despite the fact that the usage of orthobiologics in the Netherlands is low at this moment, the recent surge in research should be watched closely, because the concept holds a great potential. Future research should strive for standardization of treatment, description of indication (e.g. stage of disease) and focus on the clinical outcomes after use. Another interesting research topic would be to compare the practice variation in use between different countries. This study in combination with the ORBIT (orthobiologics Initiative) of the ESSKA (European Society for Sports Traumatology, Knee Surgery and Arthroscopy) could be a first step.

## Conclusion

Although some orthobiologic treatments might be (scientifically proven) effective and the research interest is growing, our study shows that the majority of orthopaedic and sport physician clinicians in the Netherlands still does not make use of orthobiologics as a treatment option, but almost a third of them is contemplating to start using orthobiologics.

## Supplementary Information

**Additional file 1 **: **Supplementary Table 1** – questions survey.
