# Supplementary material for: Orthobiologics and hyaluronic acid usage in the Netherlands: an electronic survey of 265 orthopaedic surgeons and sports physicians
Source: J Exp Orthop. 2021 Aug 19;8:66. doi: 10.1186/s40634-021-00380-9 (PMC8377114; doi:10.1186/s40634-021-00380-9)
Supplement: Supplementary file 1 — Additional file 1 : Supplementary Table 1 – questions survey. [file 40634_2021_380_MOESM1_ESM.docx]

| **Supplementary table 1 – questions survey** | |
| --- | --- |
| **Question** | **Answer options** |
| 1. What is your profession? | 1. Orthopaedic surgeon  2. Orthopaedic surgeon in training  3. Sport physician  4. Sport physician in training  5. Other (needs specification) |
| 2. How often do you treat a patient with or referred a patient for treatment with orthobiologics | 1. Daily  2. Weekly  3. Monthly  4. Yearly  5. Never |
| *3. How do you use orthobiologics? | 1. During surgery  2. As conservative treatment  3. Other (needs specification) |
| *4. What type of orthobiologics do you use? | 1. Autologous blood injection (ABI)  2. Platelet-rich plasma (PRP)  3. Autologous conditioned serum (ACS)  4. Mesenchymal stem cells (MSC)  5. Hyaluronic acid  6. Other (needs specification) |
| *5. What dosage do you use orthobiologics? | Open ended question per type of orthobiologics |
| *6. In what frequency do you use orthobiologics? | Open ended question per type of orthobiologics |
| *7. What are the indications for the use orthobiologics in the shoulder? | 1. During cuff repair  2. Rotator cuff tendinopathy  3. Other (needs specification) |
| *8. What are the indications for the use orthobiologics in the elbow? | 1. Lateral epicondylitis  2. Medial epicondylitis  3. Ulnar collateral ligament laesions  4. Biceps tendinopathy  5. Other (needs specification) |
| *9. What are the indications for the use orthobiologics in the hip? | 1. Hip osteoarthritis  2. Other (needs specification) |
| *10. What are the indications for the use orthobiologics in the knee? | 1. Knee osteoarthritis  2. Jumper’s knee  3. During ACL reconstruction  4. Other (needs specification) |
| *11. What are the indications for the use orthobiologics in the foot? | 1. Achilles tendinopathy  2. Fasciitis plantaris  3. During Achilles tendon repair  4. Conservative treatment of Achilles tendon rupture  5. Other (needs specification) |
| *12. Are there other indications for the use orthobiologics? | 1. No  2. Yes (needs specification) |
| *13. What is/are the reason(s) for the use of orthobiologics? | 1. Effective treatment  2. Postponing surgical treatment  3. Placebo effect  4. Other (needs specification) |
| *14. Do you receive help making or administering the orthobiologics? | Open ended question per type of orthobiologics |
| *15. Are you satisfied of the results of treatment with orthobiologics? | 1. Very satisfied  2. Satisfied  3. Neutral  4. Unsatisfied  5. Very unsatisfied |
| 16. What is the most important reason for NOT using orthobiologics? | 1. Lack of scientific evidence  2. Lack of good experience  3. Other (needs specification) |
| 17. Are you contemplating to start using or increase the use of orthobiologics in the future? | 1. Yes  2. No |
| 18. Any additional remarks? | Open ended question |
| * Questions only for the respondents who treat with or refer patients for treatment with orthobiologics. | |
